# An early analysis of the cost-effectiveness of a diagnostic classifier for risk stratification of haematuria patients (DCRSHP) compared to flexible cystoscopy in the diagnosis of bladder cancer

**DOI:** 10.1371/journal.pone.0202796

**Published:** 2018-08-23

**Authors:** Andrew J. Sutton, John V. Lamont, R. Mark Evans, Kate Williamson, Declan O’Rourke, Brian Duggan, Gurdeep S. Sagoo, Cherith N. Reid, Mark W. Ruddock

**Affiliations:** 1 Test Evaluation Group, Institute of Health Sciences, University of Leeds, NIHR Diagnostic Evidence Cooperative Leeds, Leeds, United Kingdom; 2 Randox Laboratories Ltd, Molecular Biology, Crumlin, County Antrim, Northern Ireland; 3 Department of Urology, Belfast City Hospital, Belfast, Northern Ireland; 4 Centre for Cancer Research and Cell Biology, Queen's University Belfast, Belfast, Northern Ireland; 5 Department of Pathology, Belfast City Hospital, Belfast, Northern Ireland; 6 Ulster Hospital Dundonald, South Eastern Trust, Belfast, Northern Ireland; Ottawa Hospital Research Institute, CANADA

## Abstract

**Background:**

Urothelial bladder cancer (UBC) is the 5^th^ most common cancer in Western societies. The most common symptom of UBC is haematuria. Cystoscopy the gold standard for UBC detection, allows direct observation of the bladder, but is expensive, invasive, and uncomfortable. This study examines whether an alternative new urine-based diagnostic test, the DCRSHP, is cost-effective as a triage diagnostic tool compared to flexible cystoscopy in the diagnosis of UBC in haematuria patients.

**Methods:**

A model-based cost-utility analysis using cost per quality adjusted life year and life year gained, parameterised with secondary data sources.

**Results:**

If the DCRSHP is targeted at haematuria patients at lower risk of having bladder cancer e.g. younger patients, non-smokers, then it can be priced as high as £620, and be both effective and cost-effective. Sensitivity analysis found that DCRSHP is approximately 80% likely to be cost-effective across all willingness to pay values (for a QALY) and prevalence estimates.

**Conclusion:**

This analysis shows the potential for a non-invasive test to be added to the diagnostic pathway for haematuria patients suspected of having UBC. If the DCRSHP is applied targeting haematuria patients at low risk of UBC, then it has the potential to be both effective and cost-effective.

## Background

Urothelial bladder cancer is the 5^th^ most common cancer in Western societies and accounts for 10,000 new cases in the UK and 180,000 in the EU [1]. The most common symptom of bladder cancer is blood in the urine (haematuria), which is usually painless. Haematuria can be visible to the patient (macroscopic) or non-visible (microscopic) which is detected following a routine urine dipstick test. Approximately 15% to 22% of patients presenting with visible haematuria and 2% to 11% of those with non-visible haematuria have bladder cancer [2]. Although haematuria is a strong predictor of bladder cancer [3, 4] many of its causes are benign and this creates uncertainties for primary care physicians which impacts on the consistency of their decisions around investigations and referrals [5]. Optimal risk stratification would ensure that high risk patients presenting with haematuria were referred expediently for investigations including cystoscopy (endoscopy of the urinary bladder), cytology (which examines the appearance of cells in voided urine), and imaging of their urinary tracts, to screen for bladder cancer.

Cystoscopy—the gold standard for bladder cancer detection [6], allows direct observation of the bladder, but is invasive and uncomfortable for the patient. Cystoscopy does not allow for upper track visualisation, does not always detect small areas of carcinoma *in situ*, can give false positive results, is embarrassing for the patient, and can be biased by the risk category of the patient [7]. Cytology, has high specificity, but poor sensitivity, and hence, cannot act alone for the diagnosis of urothelial cancer [8].

There is a significant cost associated with investigating haematuria, in large part due to the cost of the cystoscopy. It has been estimated in the UK that investigating haematuria patients, found not to have bladder cancer, contributes to one third of the total cost of managing patients with non-muscle invasive bladder cancer (estimated to be £100 million in 2008) [9]. Therefore, there is a strong clinical need for an inexpensive diagnostic test. Patients that present to haematuria clinics are a heterogeneous group with respect to their risk of bladder cancer. Following their investigations, the causes of haematuria range from no identifiable cause, through infections and benign causes to urological cancers [10]. For example older patients and those from specific at-risk groups (e.g. smokers, etc.) will have an increased prevalence of bladder cancer, while younger patients (<40 years) and those with limited risk factors (e.g. non-smokers) will be at less risk [7]. However, the latter would impact negatively on women who present with advanced stage bladder cancer [5]. Unfortunately, older patients and females have longer diagnostic intervals for bladder cancer [11]. Importantly, delays in referral of high risk haematuria patients can impact on patient survival and morbidity [12, 13].

Randox in collaboration with Queens University Belfast (QUB) and hospitals in Northern Ireland have identified a diagnostic classifier for risk stratification of haematuria patients (hereafter referred to as DCRSHP). The DCRSHP is a urine-based diagnostic test that is non-invasive, rapid, and easy to use and interpret results [10]. The DCRSHP test allows high-throughput screening of the levels of protein biomarkers in patient samples using Randox’s patented biochip technology [14], and could be used to significantly reduce the number of ‘low-risk’ patients that would otherwise be scheduled for a cystoscopy. As such, use of the DCRSHP would improve waiting times for haematuria patients who require more immediate access to diagnostic services, i.e. those patients deemed at ‘high’ risk. Currently research is still at an early stage, and the test accuracy for the detection of bladder cancer in haematuria patients has only been informed by one study [10]. Furthermore, the price of DCRSHP test has yet to be decided.

Using a decision analytic model, the objective of this study was to examine the cost-effectiveness of the DCRSHP biochip as a triage diagnostic test compared with flexible cystoscopy in the diagnosis of bladder cancer in haematuria patients. Given the early stage of its product development, this analysis focuses heavily on parameter uncertainty, and seeks to provide insights into the circumstances under which the DCRSHP would be effective in terms of health gain and cost-effectiveness using early cost-effectiveness methodology [15, 16].

## Methods

This model-based economic evaluation is a cost-utility analysis adopting a health-care provider perspective in a secondary setting. The model was parameterised using secondary data sources.

### Model structure

A Markov model was developed using TreeAge Pro 2001 software (TreeAge Software Inc., Williamstown, MA, USA). Given that recurrent events can occur on the patient pathways (such as a patient experiencing a recurrence of a non-muscle invasive bladder tumour), a Markov model approach was felt to be the most appropriate. Half-cycle correction was adopted.

**Fig 1:**
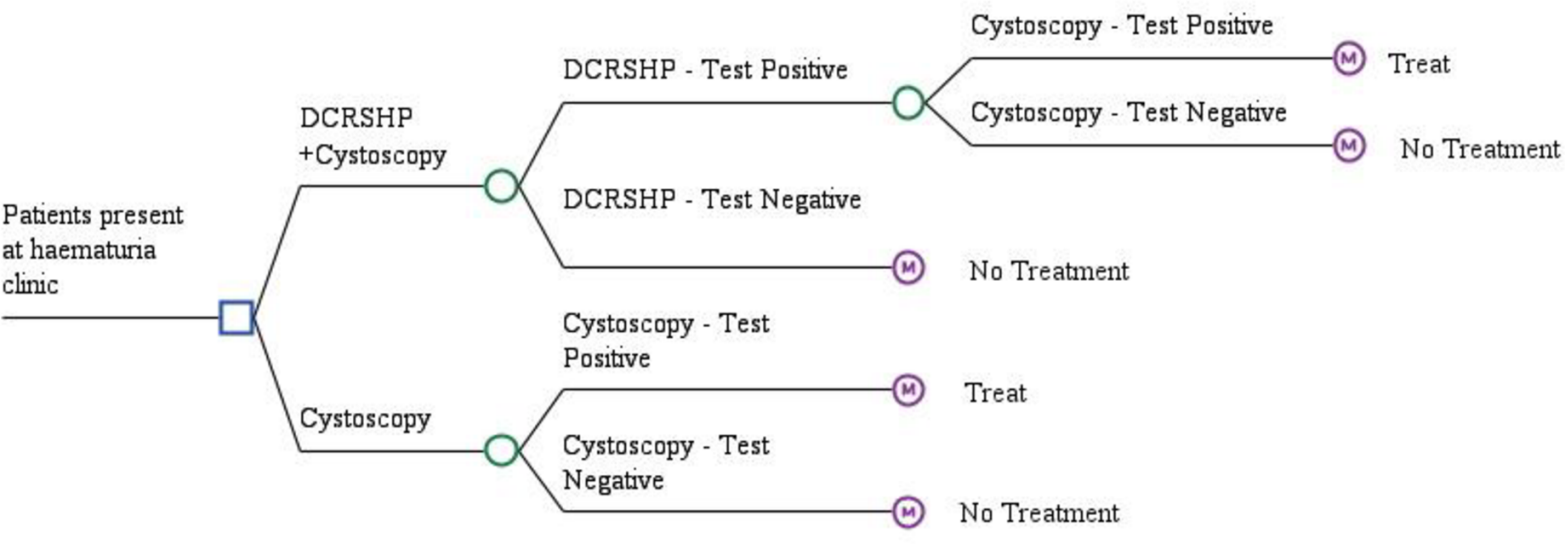
Testing pathways for the DCRSHP and usual care (cystoscopy).

Patients enter the model having presented at the haematuria clinic with non-visible or visible haematuria. Two different diagnostic pathways were compared which describe alternative approaches to the testing and diagnosis of these patients for bladder cancer. The testing pathways are shown in Fig 1:

The two arms considered in this analysis (Fig 1) are defined as follows:

- DCRSHP & flexible cystoscopy (referred to as DCRSHP)–Patients are offered the non-invasive DCRSHP biochip panel test. If this test gives a positive result, then patients move on to the standard diagnostic of flexible cystoscopy. If this is positive, patients are then offered a trans-urethral resection of bladder tumour (TURBT). Patients that receive a false positive test from both investigations will receive TURBT unnecessarily. Patients that receive a false negative test from either the DCRSHP biochip panel or flexible cystoscopy will not immediately be given treatment when they need it, but will subsequently be identified and treated over the next two years. This is to reflect that symptoms of haematuria are likely to continue and prompt reinvestigation. Amongst false negative patients, no literature is available to inform the actual number of cases that are eventually diagnosed and treated, so assumptions made by Mowatt *et al*. [17] were utilized.
- Flexible cystoscopy–Patients receive the current diagnostic standard of flexible cystoscopy. Those that test positive receive a TURBT and those that test negative receive no treatment. False negative and false positive patients are handled the same as the above pathway.
  In both cases patients are followed for the next 5 years so that the impact of the testing pathway on costs and health outcomes can be estimated.

**Fig 2:**
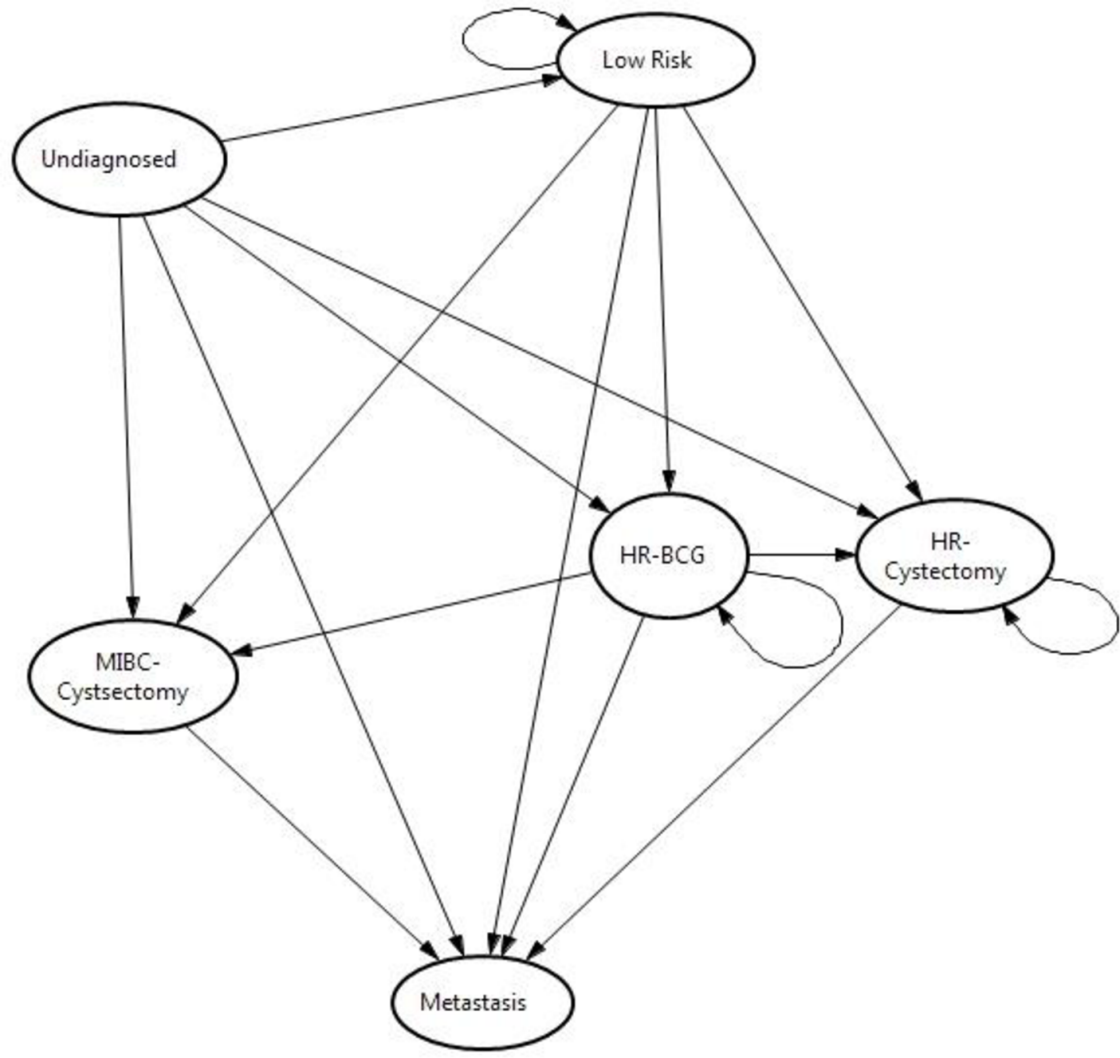
Model of treatment for bladder cancer following false-negative test result.

Fig 2 denotes the model structure for persons with a false negative result. These patients all start in an undiagnosed state and as mentioned previously will be identified correctly and moved into a cancer state over the next two years. The cancer states used within this model are low risk (LR) non-muscle invasive bladder cancer (NMIBC), high risk (HR) NMIBC-BCG (Bacille, Calmette, Guerin), HR NMIBC-Cystectomy, MIBC Cystectomy and Metastasis and represent a simplified version of the treatment and follow up strategies used in bladder cancer.

All patients receive a TURBT when transitioning from all states with the exception of HR-NMIBC Cystectomy, MIBC Cystectomy or Metastasis. The arrows indicate the transitions that occur when a patient recurs or progresses. Notably (LR) NMIBC and (HR) NMIBC can recur and remain in the same group or progress into a different state, whilst any patient who has received a cystectomy can only progress to the metastasis state. Patients from all states may experience bladder cancer related mortality, or mortality from other causes (dead state not shown in Fig 2).

The model for the true positive test status is the same as Fig 2 except it lacks the undiagnosed state and patients start immediately in a cancer state following a TURBT. Patients without bladder cancer can exist in a well or dead state with the false positive patients receiving a TURBT with negative histology.

### Model assumptions and parameterisation

The parameterisation of this model was undertaken making extensive use of secondary data sources. Given that this is an early economic evaluation, a range of values describing the price of DCRSHP and the prevalence of bladder cancer amongst patients presenting in haematuria patients were examined.

The parameters used in this model can be broadly categorized into estimates of prevalence, test accuracy parameters, transition probabilities between states, costs, and utility values. These are described below:

#### Prevalence of bladder cancer

Three previous studies were identified that informed the prevalence of bladder cancer amongst all patients presenting at haematuria clinics. These are summarized in Table 1:

**Table 1:** Reported prevalence of bladder cancer in haematuria patients.

*Study*	*Patient group*	*Prevalence of bladder cancer*
[3]	1930 patients who attended a haematuria clinic with microscopic (982) and macroscopic (948) haematuria	11.9% (230/1930)
[18]	4020 patients who attended haematuria clinic with microscopic (1949) and macroscopic (2071) haematuria	12.1% (485/4020)
[8]	778 consecutive patients attending a hospital haematuria rapid diagnosis clinic (Breakdown of micro- macro-haematuria not reported)	20% (156/778)
	**Total**	**12.95% (871/6728)**

As shown in Table 1 the patient groups in these 3 studies appear very similar and thus their values have been aggregated to provide a summary value that informs the prevalence of bladder cancer amongst haematuria patients. However, given that the DCRSHP could be applied to sub-sections of patients at more or less risk of bladder cancer, the impact of this parameter on model results was subject to sensitivity analysis.

#### Test accuracy

Although the DCRSHP test provides a risk stratification score for patients at the haematuria clinic, in this analysis the test is considered to provide either a positive or negative test result. This approach is equivalent to a risk stratification score applied to a cut-off, and allows the application of sensitivity and specificity values to be considered in the analysis.

The sensitivity and specificity of the DCRSHP test were taken from a single study [10]. For flexible cystoscopy, following a detailed search of the literature, only one study was found that describes the test accuracy for diagnosing haematuria patients with bladder cancer [8]. The test accuracy parameters used in this analysis are shown in Table 2. A description of their application in the model are given in the S1 File.

**Table 2:** Test accuracy parameters for DCRSHP and flexible cystoscopy.

Parameter	Value (95% CI)	Reference
*Flexible cystoscopy*		
Sensitivity	0.980 (0.942–0.996)	(145/148) [8]
Specificity	0.938 (0.916–0.956)	(562/599) [8]
*DCRSHP Biomarker*		
Sensitivity	0.905 (0.804–0.964)	(57/63) [10]
Specificity	0.795 (0.635–0.907)	(31/39) [10]

#### Transition probabilities

Parameters describing the probability of events related to the natural history of bladder cancer are given in Table 3. These have been informed by secondary data sources with extensive use made of the references used to parameterise the economic model implemented in the HTA report by Mowatt et al. [17].

**Table 3:** Six month model probabilities.

*Parameter*	*Value*	*Source*
Proportion of patients enter LR NMIBC state following diagnosis	105/192	Patient data—Belfast City Hospital, the Ulster Hospital Dundonald and the Craigavon Area Hospital in Northern Ireland
Proportion of patients at diagnosis with NMIBC HR that require BCG	47/192	“”
Proportion of patients at diagnosis with NMIBC HR that have cystectomy	13/192	“”
Proportion of patients with MIBC at diagnosis	14/192	“”
Proportion of patients with metastasis at diagnosis	13/192	“”
LR NMIBC probability of experiencing a recurrence	0.0638	(95% CI: 0.0622–0.0654)
HR NMIBC (BCG) probability of experiencing a recurrence	0.1393	(95% CI: 0.1368–0.1417)
Progression to MIBC from HR(BCG)	0.030	20/80 at median time 26.7 months [19]
Progression to metastasis from HR(BCG)	0.010	5/80 at median time 18.2 months [19]
Progression to Metastasis from HR(Cystectomy)	0.030	18/72 median time 25.9 months [19]
BC related death (amongst Well, BCG, and Cystectomy)	0.0048	74/1529 (Weighted averaged at 5 years [20]
BC related death MIBC	0.0497 (0-5yrs) 0.0327 (5yrs+)	Out of 1054 patients 60% survival at 5 years, 43% survival at 10 years [21]
MIBC progression to metastasis	0.0771 (0-5yrs) 0.0060 (5yrs+)	Out of 1054 patients, 32% progression at 5 years [21]
BC related mortality metastasis	0.236	5-year survival 6.8% (13/192) [22]
Proportion recurrence require Cystectomy	9/52	[23]
Cystectomy related mortality	96/4484	30d mortality data from 2008–2010 [24]
Proportion Male	0.796	(619/778) [8]
All-cause mortality	Variable by age	Office of National Statistics–mortality rates

Further parameters utilized in the decision model are shown in Table 4. Of particular note is the relative risk (RR) for progression amongst patients that have had a false negative test result, and as such their disease status is undetected. As described by Mowatt et al., [17], there are no studies that have compared survival with and without TURBT, and so the approach utilized by Mowatt et al., [17] to describe outcomes amongst this patient group has been adopted here. “Using information from the Millian-Rodriguez and colleagues’ study it was assumed that the base-case RR for progression comparing no treatment (no TURBT) with treatment (TURBT) was 2.56, that is the RR compared TURBT plus BCG with TURBT alone” [17].

**Table 4:** Additional parameters used in economic model.

*Parameter*	*Value* *(95% CI)*	*Source*
Mortality rate of TURBT	0.8% (0.3%-1.3%)	10/1250 [25]
False negative: probability detected in the first year	50%	Assumption [17]
False negative: probability detected in second year	75%	“”
False negative: probability detected after second year	100%	“”
RR for progression (no treatment vs treatment) Applied to: Progression to MIBC Progression to Metastasis BC related mortality	2.56	“” [20] Assume that SE = 1

#### Cost and resource use data

All costs used in this analysis are in UK pounds sterling £ (2014 value). NHS References costs (2014/15) were used to attribute costs to the resource use, with cost values also taken from Mowatt et al., [17] where necessary. The resource use costs used in this study are given in Table 5.

**Table 5:** Breakdown of the costs that were used in the economic analysis.

*Parameter*	*Base Case*	*Source*	*Notes*
Cystectomy	£9,816	NHS reference Costs—LB 39C/D Cystectomy with urinary diversion and deconstruction	Weighted average of LB39C and LB39D
TURBT	£2,435	[17]	Inflated from 2006 price
Palliative treatment	£160.46	NHS reference costs—SD02A Inpatient specialist palliative care, Same day 19 years and over	
Chemotherapy (cisplatin)	£50.22	British National Formulary (Sept 2016) 100mg/100ml solution for infusion vials	
CT Scan	£395	Abdomen pelvis	Inflated from [17]
Flexible Cystoscopy	£537	[17]	Inflated from 2006 price
BCG 1 vial	£71.61	British National Formulary (Sept 2016) 1 vial 12.5mg OncoTICE	

#### Utility values

The utility values used in this study were taken from three studies [26–28]. Kulkarni (2007) [27] utilized the Tufts-New England Centre Cost-effectiveness analysis registry for comparable health state preferences from populations with similar health issues. Kulkarni (2009) [26] extrapolated utility scores from other conditions in which similar health states could be expected. Stevenson (2014) [28], extrapolated some values from studies involving patients with similar conditions and complications (see Table 6).

**Table 6:** Utility values (and associated probabilities) used to inform QALY values.

*Description*	*Duration*	*Parameter*	*Reference*
*Utilities*			
Cystoscopy	6 months	0.997 (0.95 to 1) Done	[27] applied over 6-month time step
TURBT	7d	-0.1 (SE = 0.02)	[26]
BCG Induction	6 weeks	-0.02 (-0.3 to 0.0)	[27]
BCG Complications	6 months	-0.2 (-0.4 to 0)	[27]
Cystectomy	60d	0.8 (0.5 to 1)	[27]
Pre-diagnosed utility		0.78 (0.52–1.0)	Assumed by [17]
Impotence after cystectomy utility	Permanent	0.91 (0.69 to 1)	[27]
Post-cystectomy State	“”	0.96 (0.72 to 1)	[27]
Cystectomy short term complications	60d	-0.3 (-0.6 to 0.0 assumed)	[27]
Chemotherapy	103d	-0.36 (-0.9 to -0.2)	[27, 28]
Metastases responsive to chemotherapy	Permanent	0.62 (0.31 to 0.93)	[27]
Metastases unresponsive to chemotherapy	Permanent	0.3 (0.13 to 0.62)	[27]
*Probabilities*			
BCG Complication probability		0.286 (0.18 to 0.67)	[27]
Post-cystectomy probability of short term complication		0.267 Range 0.2–0.304	[27]
Probability of Impotence after cystectomy		0.59 Range 0.33–1.0	[27]
Probability of response to chemotherapy for metastatic cancer	“”	0.425 Range 0.381–0.57	[27]

#### Model assumptions

As part of the modelling framework, it was necessary to make a number of assumptions to enable this analysis to be carried out. These are listed as follows:

- Patients in the LR NMIBC state receive a cystoscopy at 3 months and then annually for 5 years
- HR NMIBC BCG patients receive 3 vials of BCG every 3 months for 3 years. A cystoscopy is performed every 3 months for 2 years, every 4 months in the 3rd year and annually thereafter. Annual CT urogram is performed.
- (HR) NMIBC Cystectomy have a cystectomy followed by a followed by a CT abdomen pelvis every 4 months for 1 year, 6 monthly for 2 years and annually thereafter.
- MIBC patients are well enough to receive a cystectomy.
- MIBC patients have 3 cycles of chemotherapy and a cystectomy followed by a CT abdomen pelvis every 4 months for 1 year, 6 monthly for 2 years and annual thereafter.
- Metastatic patients do not receive palliative TURBT or radiotherapy.
- Metastatic patients are responsive to chemotherapy and receive 3 cycles every six months.
- Metastatic patients receive 135 days of palliative care [1]
- All patients accept the tests and treatments offered.
- Undiagnosed patients are at increased risk of MIBC and metastasis (see below).
- For patients that test false negative, 50% are identified in the first year, 75% are identified by the second year, and the remainder are identified after the second year. [1]
- Patients are 60 years old.

#### Analysis

This model-based economic evaluation utilizes the primary outcome of the cost per quality adjusted life year (QALY). A time step of 6 months was applied in the Markov model with a 5-year time horizon. This time horizon was chosen since it provides a more stringent examination of the DCRSHP test, as it does not allow more uncertain benefits that occur many years in the future to be included. Discounting was applied at 3.5% for costs and outcomes as recommended by NICE [29]. The results are presented using the incremental cost-effectiveness ratio (ICER) which is defined as the difference in the costs of the two strategies divided by the difference in their outcomes, and net-monetary benefit (NMB) which is defined for each intervention as:

NMB=QALYsgainedxwillingnesstopay(WTP)foraQALY–Costoftheintervention.

Given that the utility values were not obtained using a robust theoretical approach, results utilizing the life year outcome at baseline were also reported.

#### Sensitivity analysis

This analysis contains a number of important uncertainties that must be examined. These were examined through one-way, two-way, and probabilistic sensitivity analyses.

1. Given this is an early economic evaluation it is necessary to gain insights into the price of the DCRSHP test. The price of the DCRSHP test was varied to show the point at which the cost of the test leads to an ICER of £20,000/QALY which is at the low end of the threshold for acceptance of an intervention as given by NICE [29].
2. The prevalence of bladder cancer in the patient population presenting with haematuria is an important factor in this context as the DCRSHP could be used to target specific sub-groups of the haematuria patient population. Thus sensitivity analysis was conducted in order to show the cost-effectiveness of the DCRSHP at a range of plausible prevalence values.
3. Given the invasive nature of the cystoscopy, its impact on quality of life was examined across a range of plausible values.

Probabilistic sensitivity analysis (PSA) was implemented by using Beta distributions where data made this possible, using the method of moments to obtain the Alpha and Beta parameters in each case [30]. Where a range was described for parameter uncertainty as was the case for utilities and their associated probabilities, then the standard error for the Beta distribution was estimated as follows:

$$SE=\frac{U-L}{2\times 1.96}$$

Where U and L are the upper and lower limits of the range respectively [31]. From this, the alpha and beta to inform the beta distribution were calculated from the following:

$$Mean=\frac{\alpha }{\alpha +\beta }\;sd=\sqrt{\frac{\alpha \beta }{{\left(\alpha +\beta \right)}^{2}\left(\alpha +\beta +1\right)}}$$

For decrements in the utility values, log-normal distributions were used.

#### Expected value of information

Using a nonparametric regression-based method that requires only a probabilistic sensitivity analysis sample (i.e., in this case 10,000 samples drawn from the joint distribution of the parameters and the corresponding net benefits), expected value of perfect information (EVPI) and the expected value of perfect parameter information for single and groups of parameters was calculated [32] (see S1 File).

## Results

### Examination of the price of the DCRSHP test

**Fig 3:**
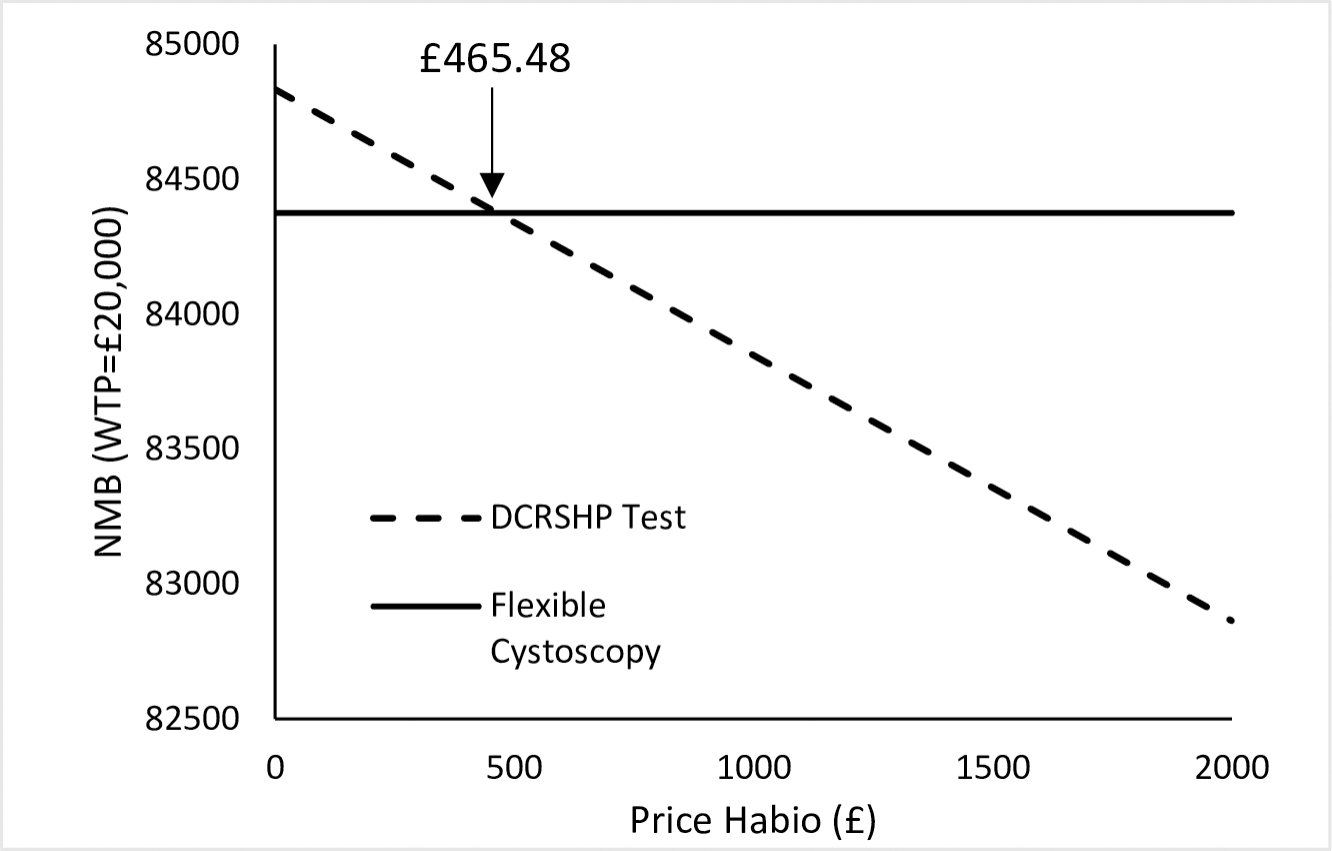
Net Monetary Benefit values for flexible cystoscopy and the DCRSHP test with variations in the cost of the DCRSHP test at a WTP of £20,000 for a QALY.

Taking all parameters at baseline, and using NMB for each intervention at a WTP for a QALY of £20,000, the NMB for the DCRSHP test and flexible cystoscopy with variation in the price for the DCRSHP test is shown in Fig 3. As shown in Fig 3, at a WTP of £20,000 for QALY the DCRSHP test can be priced up to £465 and still be cost-effective versus flexible cystoscopy. The results for the DCRSHP test priced at £465.48 are shown in Table 7. Utilizing an overall prevalence of 12.95% for bladder cancer for all haematuria patients who present at haematuria clinics, the DCRSHP test is more cost-effective than flexible cystoscopy at a willingness to pay for a QALY of £20,000 (DCRSHP priced at £465.48). The DCRSHP test is marginally more effective than flexible cystoscopy in terms of QALYs gained, and is more effective in terms of life years gained. A simple budget impact analysis examining the impact of varying the price of the DCRSHP test on a budget is described in the Appendix.

**Table 7:** Cost-effectiveness output for the DCRSHP test = £465.48 using the outcome of the QALY.

Strategy	Cost	Cost diff.	Effect (QALYs)	diff. (QALYs)	ICER (Cost/QALY)	Effect (LYGs)	Diff (LYG)	Proportion additional BC cases diagnosed
Flex. Cystoscopy	£1,903		4.3139			4.4377		0.0114
DCRSHP Test	£1,904	0.76	4.3140	0.0001	£20,088	4.4390	0.013	

(QALY–quality adjusted life year, LYG–life year gained, BC–Bladder Cancer)

Note: ICER for QALY not exactly £20,000 due to a rounded value of the price for the DCRSHP test being used

### Prevalence

**Fig 4:**
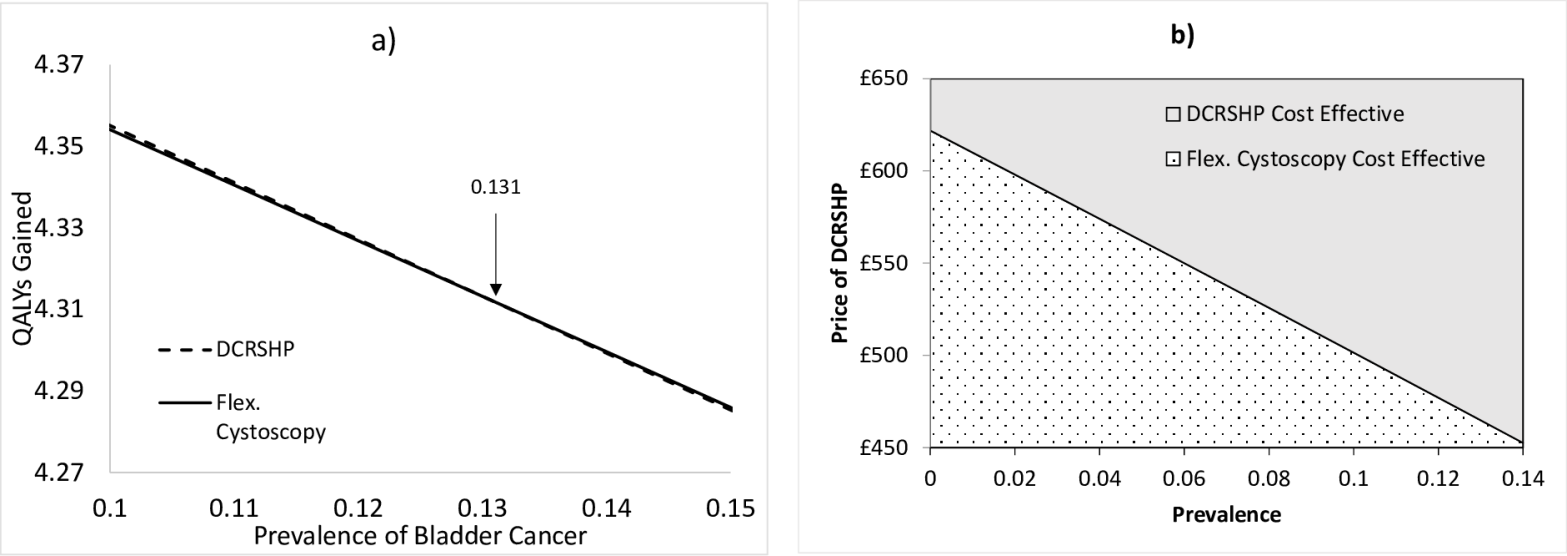
a) Sensitivity analysis of prevalence of bladder cancer for QALYs gained for the DCRSHP test and flexible cystoscopy; and b) Two-way sensitivity analysis of price of the DCRSHP test versus prevalence of bladder cancer at a willingness to pay for a QALY of £20,000.

As shown in Fig 4A, the DCRSHP test is more effective in terms of QALYs gained than flexible cystoscopy at a prevalence of bladder cancer of up to 0.14 after which flexible cystoscopy becomes more effective. Thus to maximize its effectiveness the DCRSHP test should be used for haematuria patients at low risk of having bladder cancer. Adopting a range of prevalence values at which the DCRSHP test is more likely to be effective than flexible cystoscopy in terms of QALYs gained, it can be seen (Fig 4B) that the DCRSHP test can be priced from £450 to approximately £620 depending on the prevalence and be more cost-effective than flexible cystoscopy.

### Probabilistic sensitivity analysis—Results

Using a price range for the DCRSHP test of £50 to £620, the cost-effectiveness plane and cost-effectiveness acceptability curve for the probabilistic sensitivity analysis are shown in the Appendix. The results show that at a threshold value for cost-effectiveness of £20,000 per QALY, DCRSHP has a probability of 0.68 of being cost-effective. And the DCRSHP test is always more likely to be cost-effective across all WTP thresholds for the QALY up to £100,000.

## Discussion

Using a model-based economic evaluation with the outcome measures of the QALY and life years gained, this study has examined the cost-effectiveness of using the DCRSHP as a new triage test for patients presenting at haematuria clinics, and compared this to the usual strategy of giving a flexible cystoscopy to all patients.

The results indicate that if the DCRSHP test were administered to all patients, then it can be priced at up to £465 and still be cost-effective at a WTP for a QALY of £20,000. In this case DCRSHP is more effective than flexible cystoscopy in terms of QALYs and life years gained (QALY: 4.3140 vs. 4.3139; LYG: 4.4390 vs. 4.377). Examining the impact of the prevalence of bladder cancer in haematuria patients it was found that the DCRSHP test is more effective than flexible cystoscopy in terms of QALYs gained up to a value of 0.131 above which flexible cystoscopy becomes more effective. This indicates that the DCRSHP test is more effective when targeting slightly lower risk sub-groups. Varying both the price of the DCRSHP test and prevalence (in the range 0 to 0.14) demonstrated that the DCRSHP test is likely to be more cost-effective and effective than flexible cystoscopy up to a prevalence of 0.131 and up to a price of approximately £620.

The PSA revealed the impact of the uncertainty in the parameters on the model results. When adopting a price range of £50 to £620, the cost-effectiveness acceptability curve reveals the DCRSHP test is more than 55% likely to be cost-effective across all willingness to pay values for a QALY of up to £100,000. Although it is noted that the price of the DCRSHP test can be selected and the prevalence of the targeted patient population can be strongly influenced by selecting younger patients with fewer risk-factors, thus reducing the uncertainty considered in this analysis.

The strengths of this analysis are that the best available evidence has been used to parameterise a full model-based economic evaluation. As this is an early model, extensive sensitivity analysis has been conducted to identify the impact of key parameters on the conclusions drawn from the model. We believe that such analyses are a key component of early cost-effectiveness analyses [16]. Nonetheless a number of limitations are acknowledged. The use of the QALY outcome measure relied on the use of utility data which had not been collected using a validated approach. Although this uncertainty was reflected in the distributions used in the PSA, this shortcoming is noted. Moreover, given that this is an early economic evaluation, the price of the DCRSHP test had not been set, and the parameters describing its sensitivity and specificity relied on relatively small data points. The uncertainty of these values was acknowledged and analysed in the sensitivity analysis, however their shortcomings are further noted here.

In a previous HTA report Mowatt et al., [17], the cost-effectiveness of a number of non-invasive tests in the diagnosis of bladder cancer were investigated. The authors concluded that strategies incorporating flexible cystoscopy were most cost-effective in terms of true positive cases and life years gained. However, Mowatt et al., [17] did not use the QALY outcome and no attempt was made to incorporate the impact of a flexible cystoscopy on patient outcomes in their study. Here a decrease in quality of life has been has been incorporated for patients that receive the cystoscopy in the form of a slight QALY decrement [27]. Varying this parameter has a significant impact on the results of this analysis, and is the equivalent of a loss of approximately half a day of perfect health. Given the physical aspect of having to experience an invasive cystoscopy, as well as the mental aspect of waiting for and worrying about receiving a potentially positive test result, this value is plausible. However, future work should examine this parameter in more detail.

The parameters used in this analysis to describe the test accuracy of cystoscopy in detecting bladder cancer are very high, with other studies, arguing that the sensitivity and specificity of cystoscopy are actually lower, e.g. sensitivity = 71% and Specificity = 72% [17]. A consequence of this is that this analysis represents a robust test of the cost-effectiveness of DCRSHP as a triage test compared to cystoscopy alone. This analysis has demonstrated that DCRSHP is cost-effective due to the reduction in the number of invasive procedures that patients would otherwise experience. Although the sensitivity (91%) and specificity (79%) of DCRSHP are lower than those used for cystoscopy in this analysis, evolution of the DCRSHP algorithm using artificial intelligence may increase its sensitivity and specificity in the near future.

The results of this early cost-effectiveness modelling study show the potential for the addition of a non-invasive test into the current testing pathway as a triage prior to receiving a cystoscopy for the diagnosis of bladder cancer.

Currently the price of cystoscopy is over £500 and given that it is an invasive test, there is scope to add a cheaper non-invasive test to the testing pathway. However, the key issue is whether adding a new test to the existing testing pathway can have a positive impact on patient outcomes, compared to just administering flexible cystoscopy to all patients. Our preliminary results indicate that the DCRSHP test as a triage test has the potential to be more effective in terms of QALYs gained and cost-effective particularly when administered to patients who are at low risk of having bladder cancer. Future work will seek to confirm these findings through a larger study to better inform the test accuracy of the DCRSHP test, particularly in this low risk population. In addition, the impact of cystoscopy on quality of life should be investigated in an attempt to demonstrate the health benefits of an alternative non-invasive test. The results of this study in addition to focussed additional work will inform and streamline the evidence generation pathway for this new non-invasive bladder cancer diagnostic test whilst highlighting the usefulness of early cost-effectiveness modelling embedded within the test development pathway.

## Supporting information

S1 File Appendix. (DOCX)
