# Supplementary material for: An early analysis of the cost-effectiveness of a diagnostic classifier for risk stratification of haematuria patients (DCRSHP) compared to flexible cystoscopy in the diagnosis of bladder cancer
Source: PLoS One. 2018 Aug 23;13(8):e0202796. doi: 10.1371/journal.pone.0202796 (PMC6107278; doi:10.1371/journal.pone.0202796)
Supplement: S1 File — (DOCX) [file pone.0202796.s001.docx]

**An early analysis of the cost-effectiveness of a diagnostic classifier for risk stratification of haematuria patients (DCRSHP) compared to flexible cystoscopy in the diagnosis of bladder cancer**

**Appendix**

*Calculation of test accuracy parameters*

The possible resulting test statuses for patients following the DCRSHP testing pathway are shown in the Table below.

| ***Patient test status*** | ***Tests received (and their result)*** |
| --- | --- |
| True positive (TP) | DCRSHP (TP) AND Flex. (TP) |
| True negative (TN) | DCRSHP (TN) OR [DCRSHP (FP) AND Flex. (TN)] |
| False positive (FP) | DCRSHP (FP) AND Flex. (FP) |
| False negative (FN) | DCRSHP (FN) |

**S1 Table:** *Test status for patients following the* DCRSHP *+usual testing strategy (Flex. – flexible cystoscopy)*

As previously noted, patients in the DCRSHP arm first receive the DCRSHP non-invasive test, and if this is positive then the patients have a confirmatory flexible cystoscopy. For a true positive (TP) test status, patients must receive a TP DCRSHP test result and a TP flexible cystoscopy result. The other statuses follow the same logic, although it should be noted that patients that are true negative may achieve this test status from two permutations of test results.

**Results – Sensitivity Analysis**

*Impact of cystoscopy on patient outcomes*

A key advantage of adding the DCRSHP test as a triage test to the testing pathway is that patients that do not have bladder cancer can avoid the invasive cystoscopy. Evidence on the impact of the cystoscopy on quality of life is limited with just a single study [1] informing this parameter. Moreover, as previous noted, this parameter was obtained without using a validated approach. Thus, taking the price of the DCRSHP test to be £465.48, the impact of varying this parameter on the model results is shown in the Table.

| **Utility** | **STRATEGY** | **COST** | **Inc. Cost** | **Eff (QALY)** | **Inc. Effect** | **ICER (Cost/QALY)** |
| --- | --- | --- | --- | --- | --- | --- |
| 0.975 | Flex. Cystoscopy | £1,903.50 |  | 4.3039 |  |  |
|  | DCRSHP | £1,904.26 | £0.76 | 4.3079 | 0.0039 | £192 |
| 0.98 | Flex. Cystoscopy | £1,903.50 |  | 4.3062 |  |  |
|  | DCRSHP | £1,904.26 | £0.76 | 4.3093 | 0.0030 | £248 |
| 0.985 | Flex. Cystoscopy | £1,903.50 |  | 4.3085 |  |  |
|  | DCRSHP | £1,904.26 | £0.76 | 4.3106 | 0.0022 | £349 |
| 0.99 | Flex. Cystoscopy | £1,903.50 |  | 4.3108 |  |  |
|  | DCRSHP | £1,904.26 | £0.76 | 4.3120 | 0.0013 | £592 |
| 0.995 | Flex. Cystoscopy | £1,903.50 |  | 4.3130 |  |  |
|  | DCRSHP | £1,904.26 | £0.76 | 4.3134 | 0.0004 | £1,929 |
| 0.997 | Flex. Cystoscopy | £1,903.50 |  | 4.3139 |  |  |
|  | DCRSHP | £1,904.26 | £0.76 | 4.3140 | 0.0001 | £20,088 |
| 1 | Flex. Cystoscopy | £1,903.50 |  | 4.3153 |  |  |
|  | DCRSHP | £1,904.26 | £0.76 | 4.3148 | -0.0005 | Dominated |

**S2 Table:** *One-way sensitivity analysis of the parameter describing the impact of cystoscopy on quality of life*

As shown in the Table. The more negative the impact of cystoscopy on quality of life, the more cost-effective the DCRSHP test becomes. If there is no impact on quality of life of the cystoscopy, then flexible cystoscopy dominates (less costly, more effective) the DCRSHP test.

Probabilistic Sensitivity Analysis

The results indicate that the DCRSHP test is unlikely to be cost-effective when priced above £620. Therefore, the PSA was implemented to reflect this, with the price being assumed to follow a uniform distribution from £50 to £620.


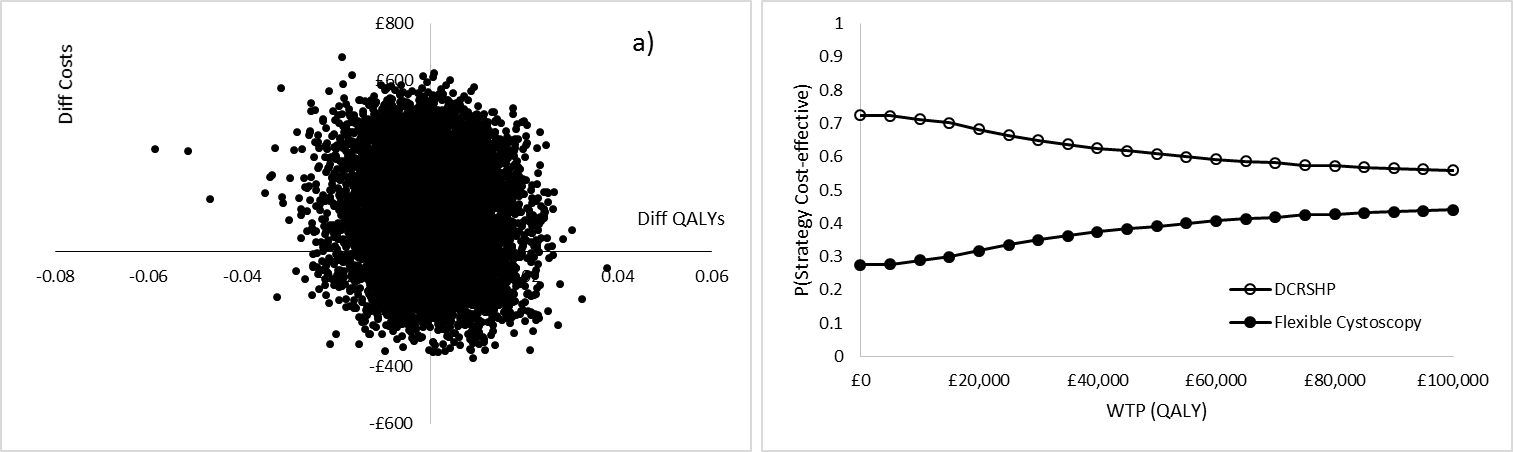


**S1 Fig.** a) *Cost-effectiveness plane and b) cost-effectiveness acceptability curve for 10,000 model iterations (price range £50 to £620)*

The results for running the model 10,000 times and sampling from distributions describing the model parameters, are shown in the Figure. The model results occupy all four quadrants of the cost-effectiveness plane suggesting that the DCRSHP test may be more or less costly than flexible cystoscopy and more or less effective in terms of QALYs gained. However, it is noted that the minority (approximately 27%) of the model results are in the southern half, suggesting that the DCRSHP test is more likely to be more expensive than flexible cystoscopy.

The Cost-Effectiveness Acceptability Curve (CEAC) shows the probability that all strategies are cost-effective at varying thresholds. The results show that at a threshold value for cost-effectiveness of £20,000 per QALY, the strategy with the highest probability of being most cost-effective is DCRSHP, with a probability of 0.68.

*Expected Value of Perfect Information - Results*

Expected value of perfect information analysis was applied over a 5-year period with discounting. Given that 193,417 cystoscopies were administered in 2015 according to NHS diagnostic test activity data, it was assumed here that 100,000 cystoscopies were administered to patients presenting annually in haematuria clinics for diagnosis.


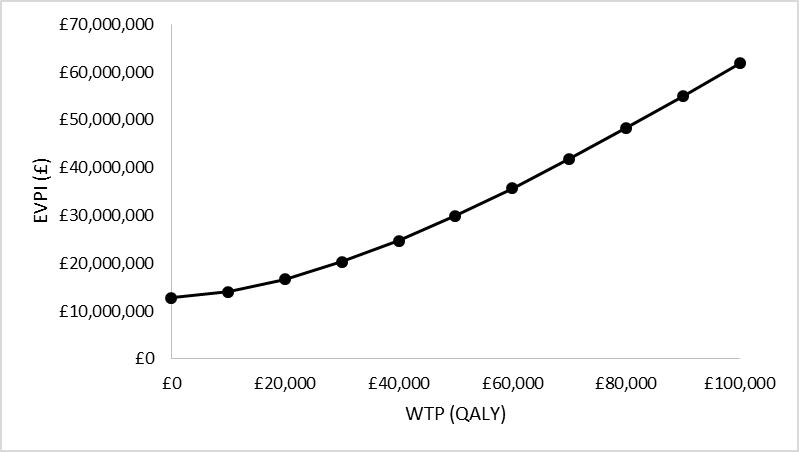


**S2 Fig.** *Value of perfect information with variation in the willingness to pay for the QALY, assuming 100,000 patients present at haematuria clinics each year for five years with a discount rate of 3.5%*

The figure above shows that the value of perfect information for WTP values of the QALY of £10,000 to £20,000 is in the range of approximately £15million to £25million.

Expected Value of Perfect Parameter Information

The expected value of perfect parameter information for single parameters is shown in the table below.

**S3 Fig.** *Single Parameter Expected Value of Perfect Parameter Information assuming 100,000 patients present at haematuria clinics each year for five years with a discount rate of 3.5% at a WTP for a QALY of £20,000*

As shown in the figure above, when considering each parameter separately, it was found that there is potential value in reducing the uncertainty of only 7 parameters, with clearly there being most value in removing the uncertainty in the price of the test.

However, it is often more informative if the EVPPI can be computed for sets of related parameters, as this can inform the maximum value of further research to jointly inform these sets of parameters. Here 4 groups of parameters were considered, these being: 1) The price and test accuracy of DCRHSP; 2) parameters describing the natural history of BC (e.g. progression rates, probability of recurrence, mortality rates etc.); 3) Utility values; and 4) characteristics of patients at presentation with haematuria (e.g. prevalence, stage of disease, gender, etc.).

*
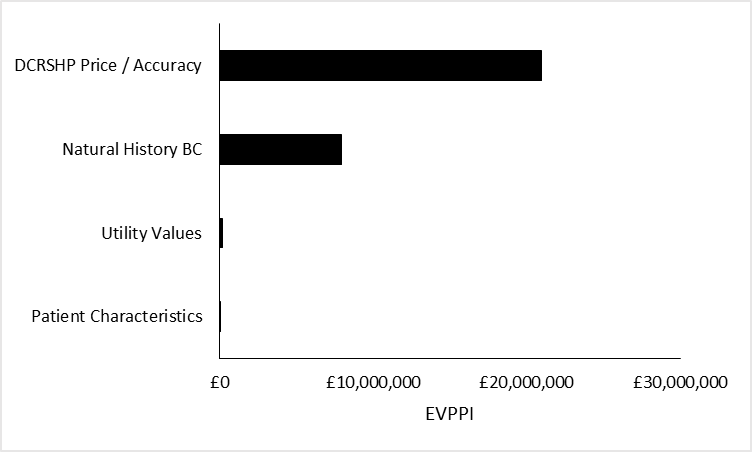
*

**S4 Fig.** *Expected value of perfect parameter information for groups of parameters assuming 100,000 patients present at haematuria clinics each year for five years with a discount rate of 3.5% at a WTP for a QALY of £20,000*

As shown in the figure, it can be seen that the EVPPI estimates are highest for the test characteristics following by the parameters describing the natural history of BC. In comparison there is very little value in conducting further research to resolve the uncertainty in the utility values and patient characteristics.

*Simple Budget Impact Analysis*

At baseline, the results here have shown that at a price of £465.48 the DCRSHP test is cost-effective compared to flexible cystoscopy with an additional incremental cost of £0.76 per patient. Given that approximately 100,000 diagnostic cystoscopies are administered in haematuria clinics in England and Wales this leads to an additional annual cost of implementing the test of £76,000. Should the price of the DCHRSHP test be lowered, then this would lead to savings that could be passed on to the NHS. Indeed, if the price of the DCRSHP test was £400, then this would lead to an incremental saving of £64 per patient, and an annual saving of £6.4million.

**References**

1. Kulkarni GS, Finelli A, Fleshner NE, Jewett MA, Lopushinsky SR, Alibhai SM. Optimal management of high-risk T1G3 bladder cancer: a decision analysis. PLoS medicine. 2007;4(9):e284. doi: 10.1371/journal.pmed.0040284. PubMed PMID: 17896857; PubMed Central PMCID: PMC1989749.
